# Perceptions about traditional Chinese medicine use among Chinese breast cancer survivors: A qualitative study

**DOI:** 10.1002/cam4.5046

**Published:** 2022-09-08

**Authors:** Yik‐Laam Hung, Siu‐Sing Leung, Siu‐Ting Pamela Chiu, Pik‐Yi Li, Anson Chi‐On Kan, Chi‐Chung Lo, Sze‐Yuen Zeta Wong, Sze‐Lam Luk, Cheuk‐Lam Charlotte Lai, Aya El Helali, Wendy Wing‐Lok Chan

**Affiliations:** ^1^ Li Ka Shing Faculty of Medicine University of Hong Kong Hong Kong; ^2^ Department of Clinical Oncology, Li Ka Shing Faculty of Medicine University of Hong Kong Hong Kong

**Keywords:** breast cancer, hormone therapy, QOL, quality of life, survival

## Abstract

**Introduction:**

An increasing number of breast cancer survivors (BCS) use traditional Chinese medicine (TCM) throughout their cancer journey. There is emerging evidence that TCM is effective in the reducing side effects of chemotherapy. However, qualitative patient‐centric and culturally relevant research into TCM use is scant. This qualitative study aimed to explore the use and perceptions of Chinese Hong Kong BCS using TCM.

**Methods:**

Participants were recruited from a university hospital and three breast cancer patient groups in Hong Kong. Questionnaires regarding the use of TCM were given to all participants, followed by individual semi‐structured interviews on selected BCS to comprehensively understand TCM's use and perceptions. A greater emphasis was placed on the qualitative data.

**Results:**

About half of the participants (*n* = 67, 48.9%) used TCM during their cancer treatment journey, among which almost all (*n* = 64, 95.5%) had improved symptoms. Sleeping disturbances (*n* = 58, 86.6%) and fatigue (*n* = 53, 79.1%) were the two most common symptoms that improved after TCM. Interview data revealed that participants used TCM to satisfy unmet needs that mainstream conventional Western medicine could not fulfil. They wished for a sense of control and better well‐being. They expressed improvements in physical and psychological well‐being after the use of TCM. Despite existing barriers, including high cost, long duration of treatment, and disapproval from oncologists, most would still recommend TCM to fellow survivors.

**Conclusions:**

Chinese Hong Kong BCS who used TCM reported positive experiences. Understanding how BCS perceive and use TCM is important to integrating TCM into survivorship care in this population.

## Graphical abstract

Over 95% breast cancer survivors who used traditional Chinese medicine (TCM) had improvement in symptoms. The most common symptoms improved were sleeping disturbance and fatigue. Despite different types of barriers like cost and long duration of treatment, most of them still recommended TCM to fellow survivors.

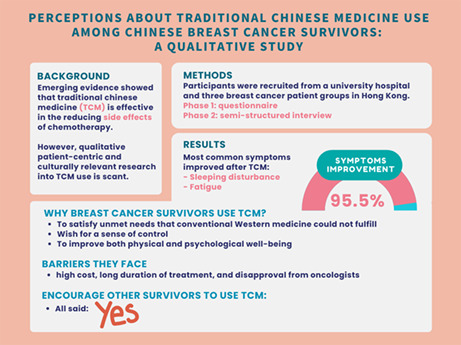

## 1 INTRODUCTION

Breast cancer (BC) has become the most common cancer in women and is the leading cause of global cancer incidence in 2020, representing 11.7% of all cancer cases.^1^ With earlier detection and advances in therapeutic interventions, overall survival and breast cancer disease‐free survival have significantly improved.^2^ Cancer survivorship among breast cancer survivors (BCS) is an important stage in the cancer paradigm. Importantly, most BCS have favourable long‐term survival rates.^3^, ^4^ Despite these improved survival outcomes, the consequences of the diagnosis of BC and the impact of treatment may result in significant physical, psychosocial, and quality of life (QOL) issues that persist throughout the survivorship phase,^5^, ^6^, ^7^ such as sleep disturbances, fatigue, depression, anxiety, and peripheral numbness.^8^, ^9^

Increasing numbers of patients use a wide range of traditional Chinese medicine (TCM), including acupuncture, Chinese herbal medicine, moxibustion, cupping, Tui Na, Qi Gong, Tai Chi, and diet modification, as an adjunct to conventional treatment.^10^, ^11^, ^12^ TCM takes a holistic approach to understand the normal body function and disease processes. Additionally, TCM focuses on both prevention and treatment.

Emerging evidence has demonstrated the favourable impact of TCM in BC treatment. A systematic review and meta‐analysis by Sun et al. showed that TCM combined with chemotherapy significantly enhanced tumour response, improved performance status, and alleviated chemotherapy‐induced toxicity in BC patients compared with chemotherapy alone.^13^ Lee et al. demonstrated that TCM lowered the risk of death in patients with advanced BC concurrent with chemotherapy.^14^, ^15^, ^16^

Despite these promising results, it is well known that TCM takes a very personalised approach, and patients' experiences are often individualised. There is also considerable heterogeneity in the perception and use of traditional Chinese medicine within Asian societies.^17^, ^18^, ^19^ While there is growing evidence to support the use and integration of TCM, there is limited understanding in the current literature of the factors influencing attitudes and use of TCM in BCS. Hong Kong is a unique city with a fusion of Eastern and Western cultures. While most BC patients have their treatment based on the Western models, beliefs regarding the role of TCM are deeply embedded within the culture.^20^ This qualitative study aims to get an in‐depth understanding of the perceptions of TCM use among BCS, including their experiences in utilising the service, perceived benefits, barriers encountered and their recommendations to other survivors.

## 2 METHODS

This study was approved by the Institutional Review Board of the University of Hong Kong/Hospital Authority Hong Kong West Cluster (HKU/HA HKW IRB).

### 2.1 Participants

Eligible participants were Chinese BCS, aged 18 or above, female, had completed anticancer treatment for at least 1 year and could read Chinese and speak Cantonese/Putonghua. Survivors who were currently on adjuvant hormonal therapy were also eligible to participate. Exclusion criteria included patients with recurrent disease or distant metastasis and patients who had other malignancies during the study.

BCS were recruited in the breast cancer clinics in the Department of Clinical Oncology, Queen Mary Hospital, a university hospital in Hong Kong, or by email invitation through three breast cancer groups in Hong Kong from November 2020 to February 2021.

### 2.2 Study design

This was a two‐phase qualitative study: phase I with a cross‐sessional questionnaire to identify BCS who used TCM and phase II with semi‐structured interviews to obtain an in‐depth understanding of the participant's perceptions of the benefits and barriers to the usage of TCM.

#### 2.2.1 Phase I—questionnaire study

Participants were invited to complete a questionnaire in Chinese. Those recruited in the breast clinics completed the questionnaire in paper format, while those recruited by email used an online questionnaire. The questionnaire was developed with a panel of clinical oncologists, nurses, public health experts, and statisticians. It was subsequently pilot tested with a group of 10 non‐healthcare workers and five cancer patients. We then used comments from these 15 individuals to revise and finalise the questionnaire.

The questionnaire consisted of five sections: (i) clinical characteristics: current age, age at diagnosis, education level, marital status, and occupation; (ii) medical history: cancer stage, type of surgery done, type of adjuvant therapy received, and experienced side effects; (iii) TCM use: period and duration of use, location of TCM clinic, and reasons for starting TCM; (iv) perceived effectiveness of TCM in relieving symptoms; and (v) difficulties faced when using TCM. The English version of the questionnaire is found in Data S1.

#### 2.2.2 Phase II—qualitative interview

Twenty‐one participants who were prescribed TCM for breast cancer management in phase I consented to undergo a semi‐structured interview in phase II.

Individual semi‐structured interviews were conducted to understand the participants' perceptions of the benefits and barriers to using TCM. Written consent was obtained when eligible BCS were asked to provide their contact information upon completion of the questionnaire. Additional verbal consent was obtained when participants were contacted and invited to attend the interview.

Three researchers conducted individual interviews in Cantonese. The interviews took place at the participant's home via ZOOM and followed all necessary confidentiality procedures. Each participant was only interviewed once, and each interview lasted 30–40 min. All interviews were audio‐recorded and transcribed verbatim for analysis. All transcripts were anonymised.

A panel of clinical oncologists and nurses developed an interview guide (Data S2). Interviewees were encouraged to share their personal stories and thoughts about their experiences with their BC diagnosis and use of TCM. Participants were sometimes asked to elaborate on certain answers.

We followed a grounded theory approach to ensure comprehensive data collection and analysis.^21^, ^22^ After every few interviews, a meeting was held with all the investigators to discuss the findings, revise the interview schedule, and select the next interviewees to facilitate the exploration of new themes as they emerged. This process is otherwise known as “theoretical sampling”.^23^ Data collection ended when no new themes emerged from the interviews, indicating that theoretical saturation was reached.^23^ Saturation was reached at 21 participants.

### 2.3 Data analysis

Data were analysed using thematic analysis with an inductive approach and enhanced with the principles of grounded theory to ensure the process was conducted systematically.^21^, ^22^, ^24^ Themes and sub‐themes were identified by adapting Braun and Clarke's six‐step thematic analysis process.^25^

The transcripts were systematically read, and initial codes were identified in a codebook. The codes were abstracted into broader categories through constant comparison within and between transcripts. These more general categories were further reviewed and refined to generate the final themes and sub‐themes. A core theme was also abstracted from the developed themes.^24^, ^26^ To summarise, transcripts were iteratively coded at open, axial and selective levels such that similar concepts were clustered and abstracted into different themes.^27^ An overarching core theme was also abstracted from all the key themes that encapsulated and contextualised all interviewees' perceptions and experiences.

This analysis process was performed by two investigators (HYL and SSL) independently. The coding manuals and theme extraction were periodically compared to ensure intercoder agreement and qualitative reliability.^28^ Interview recordings and transcripts were also cross‐checked twice to confirm a complete account of participants' responses.^28^ Discussion between the two coders and other team members resolved any discrepancies or coding uncertainties. Theoretical saturation was reached when both coders agreed that no new themes emerged from the previous few interviews.

### 2.4 Researcher characteristics & reflexivity

Qualitative research is inherently subjective. Reflexivity allows researchers to be sensitive to how their personal background and prior experiences may shape how data is collected and interpreted.^29^

All listed researchers conducted the interviews, while YL and SL conducted the analysis and interpretation. All are of Chinese ethnicity from Hong Kong, from clinical backgrounds with either medical or nursing experience, and had personal experiences with using TCM. This empowered researchers to ask culturally relevant questions, and participants may be more willing to talk more openly about their TCM experiences with Chinese people. At the same time, researchers were sensitive to how their clinical backgrounds may affect data collection and interpretation. In particular, the senior author (WL), a clinical oncologist with experience in quality of life research and breast cancer, led the interview teams, provided guidance and supported subsequent analyses.

## 3 RESULTS

### 3.1 Questionnaire

A total of 153 BCS were recruited, with 16 BCS excluded. Therefore, the final analysis included 137 participants with a median age of 55 years old (see Table 1). Participant demographics are shown in Table 1. Among the 137 participants, 57 (41.6%) participants had a survivorship of 5 years or more, 101 (73.7%) participants had received chemotherapy, 101 (73.7%) had adjuvant radiotherapy, and 77 (56.2%) had hormonal therapy. All participants had early BC, and none had advanced or metastatic BC. Among the 133 (97.1%) participants who experienced chronic symptoms after anticancer treatment, the two most common symptoms experienced were fatigue (*n* = 88, 64.2%) and sleeping disturbances (*n* = 69, 51.8%).

**TABLE 1:** Demographics of participants in phase I questionnaire study

	Number	Percentage
Total number of participants	137	100.0%
Age at study		
<40 years old	12	8.8%
40–59 years old	86	62.8%
≥60 years old	39	28.5%
Years of survivorship		
≤5 years	80	58.4%
>5 years	57	41.6%
Educational level		
Primary school or less	25	18.2%
Secondary school	55	40.1%
Tertiary school or higher	57	41.6%
Marital status		
Single/widowed	44	32.1%
Married	93	67.9%
Employment status		
Employed	75	54.7%
Unemployed/retired/housewife	62	45.3%
Treatment received		
Chemotherapy	101	73.7%
Radiotherapy	101	73.7%
Hormonal therapy	77	56.2%
Symptoms after anticancer treatment		
Fatigue	88	64.2%
Sleeping problems	71	51.8%
Psychological problems	67	48.9%
Pain	60	43.8%
Nausea	56	40.9%
Loss of appetite	48	35.0%
Memory loss	43	31.4%
Dry mouth	38	27.7%
Dizziness	38	27.7%

Sixty‐seven (48.9%) participants used TCM during their BC journey, of which 21 (31.3%) participants used TCM concurrently with anticancer treatment while 32 (47.8%) used TCM after completion of anticancer treatment. Patterns of TCM use are shown in Table 2. Herbal medicine (*n* = 67, 100%) and acupuncture (*n* = 13, 19.4%) were the two commonest TCM modalities used. 46 (68.7%) participants received information regarding TCM from family and friends, while 7 (10.4%) from healthcare professionals. To address the need for TCM, 57 (85.1%) participants wished to regulate health, 28 (41.8%) to relieve symptoms and 19 (28.3%) to prevent breast cancer recurrence. 64 (95.5%) participants felt an improvement in symptoms after using TCM. The top two symptoms that improved after TCM were sleeping disturbance (*n* = 58, 86.6%) and fatigue (*n* = 53, 79.1%). 63 (94%) would recommend TCM to other breast cancer survivors. Nevertheless, all 67 participants had difficulties using TCM. The top challenge was the time‐consuming process (*n* = 54, 80.6%).

**TABLE 2:** Pattern of use of traditional Chinese medicine (TCM)

	Number	Percentage
Total number of participants who used TCM	67	100.0%
Modalities of TCM used		
Herbal medicine	67	100.0%
Acupuncture	13	19.4%
Qi Gong	10	14.9%
Tai Chi	10	14.9%
Diet modification	9	13.4%
Tui Na	6	9.0%
Cupping	1	1.5%
External applied herbs	1	1.5%
When to start TCM?		
Long before breast cancer	7	10.4%
During anticancer treatment	21	31.3%
After anticancer treatment	32	47.8%
Where they got information about TCM?		
Recommended by friends or families	46	68.7%
Recommended by healthcare professionals	7	10.4%
Others	14	20.9%
Why they want to try TCM?		
Regulate health	57	85.1%
Prevent recurrence	19	28.4%
Relieve symptoms	28	41.8%
Perceived symptoms relieved		
Sleeping problem	58	86.6%
Fatigue	53	79.1%
Dry mouth	48	71.6%
Loss of appetite	47	70.1%
Pain	45	67.2%
Nausea	42	62.7%
Dizziness	41	61.2%
Stress or anxiety	41	61.2%
Memory loss	36	53.7%
Recommend to other breast cancer survivors?		
Highly recommend	25	37.3%
Recommend	39	60.9%
No	3	4.5%
Barriers of using TCM		
Cost	27	40.3%
Time consuming	54	80.6%
Not recommended by oncologists	20	29.9%
Lack of regulations from health system or lack of strong evidence	11	16.4%

### 3.2 Qualitative interview

There was heterogeneity in the extent of survivorship, breast cancer stage, age at diagnosis, marital status, and highest education level attained among the participants. All participants underwent surgical excision, 20 received radiotherapy, 15 received chemotherapy, 13 received hormonal therapy, and 3 received targeted therapy. All participants used herbal medicine.

The semi‐structured interviews led to the development of four main themes and a core theme which encapsulated the findings (Table 3). Several sub‐themes were identified in each theme for further discussion and are described below with supportive quotes.

**TABLE 3:** Core theme, main themes and sub‐themes identified

Core theme: Filling the care gap	
Themes	Subthemes
Accessing TCM services	Sense of Control No More Toxins Trust Built from Past Experiences and Recommendations from Others
Perceived benefits of TCM	Improving Sleep and Fatigue Relieving Side Effects of Anticancer Treatment Relieving Stress and Anxiety
Barriers encountered when using TCM	Financial Constraints Time Constraints Not Recommended by Oncologist
Recommendations on using TCM	

#### 3.2.1 Accessing TCM services

Three sub‐themes emerged for the reasons of starting TCM: (i) “sense of control”; (ii) “no more toxins”; (iii) “trust built from past experiences and recommendations from others”.

##### Sense of control

All participants mentioned that the diagnosis of BC and its treatment were a harrowing experience. Patients also highlighted that the diagnostic process, and assessments prior to the diagnosis, were overwhelming. Some mentioned they felt a lack of energy after going through the process. Multiple interviewees described a sense of helplessness before using TCM, describing it as a “storm” or an “overwhelming wave”. Participants wished to have a sense of control over their diagnosis but did not want to rely on conventional Western medicine solely.

“H” – I had to suddenly stop working … but I still had to care for my two children. … It felt like a huge overwhelming wave. … I didn't understand why all this was happening to me. I also had to cope with all these tests before the surgery, which left me quite anxious. The herbal tea could calm me down.

“N” – I felt so tired with all those investigations. It was frightening. I had a mammogram and biopsy. Then I had to wait for days to get the results. I had to go to this clinic and that clinic… waiting and waiting… I didn't want to think back on the process… too heavy for me. I wish for a control of my emotions.

##### No more toxins

Physical discomfort and psychological distress after anticancer treatment were emphasised by every interviewee. They had fatigue, difficulty in sleeping, or changes in body shape. Some believed that they had to remove the “toxins” in their body. Some even specifically mentioned that Western medicine was the “toxin” and wanted to use TCM to remove the “toxins” and regulate the body back to “normal” or alleviate the side effects.

“A” – My legs were swollen after chemotherapy. The poisons of chemotherapy were accumulating… I believed TCM could help me remove the toxins in my body. I did not want to take more Western medicine to weaken my body even further.

“B” – I felt so tired after chemotherapy. My feet felt so numb. I asked my oncologist for help, but they weren't able to. I wanted to get back to work and no longer be a burden to my family, so I tried herbal medicine…

“D” – I took herbal medicine to remove toxins. My body accumulated a lot of toxins and they prevent symptom relief if they weren't cleared.

“M” – I could not sleep well after starting hormone therapy. I did not want to rely on sleeping pills… My fingers and joints felt so stiff… I had constipation, and my stomach felt so distended…

“L” – The doctor prescribed drugs for my rash, which didn't work. It wasn't a solution, so I saw a TCM practitioner. I also didn't want to take more Western medicine.

In addition, some participants also believed that TCM was more beneficial than Western medicine in certain aspects, with its milder nature and holistic whole‐body focus.

“A” – TCM is less aggressive than Western medicine. Western medicine is poison while TCM is milder.

“E” – Even though Western medicine is quicker, TCM is milder and more comfortable. It can help ‘consolidate the foundation’ and ‘nourish the soul’.

“M” – The relieving effect of TCM lasts longer than in Western medicine.

##### Trust built from past experiences and recommendations from others

Some of the interviewees had experiences in using TCM. They used TCM regularly to manage symptoms of chronic illnesses, get rid of a cough, or regulate health. They mentioned that TCM could regulate their body and “reduce the heat and dryness” in their body. They trusted TCM would supplement anticancer therapy to help them recover and regain their health.

“C” – My family used to consult TCM. We always use herbal tea to clear away heat and toxins.

“I” – I often consult my TCM practitioner, drink herbal medicine and do acupuncture. I have nasal allergies and sometimes skin problems on my hands (homemakers' hands). I would use acupuncture on meridian points on my hands and nose.

“J” – I have always been using TCM to regulate health.

Interviewees also received recommendations and positive feedback from people surrounding them before deciding on TCM. Those mentioned included friends and family members, fellow patients in patient support groups, and even healthcare workers such as nurses who had positive experiences with TCM. They received a referral to a specific hospital or TCM practitioner. This would prompt appraisal of the interviewee's condition and the potential of TCM, regardless of their initial belief or previous experiences with TCM.

“M” – My brother had a liver problem and decided to use TCM, so he suggested I use TCM as well.

“E” – My friend who had cancer went to XX University and said the TCM practitioner there was very good, so I followed her.

“I” – The clinic nurse asked whether I believed in TCM… I would usually see a Western doctor, but after some thought, I thought it was a good idea, so I followed her advice and used Western medicine and TCM simultaneously.

“F” – My friend in the cancer support group mentioned they would see the TCM practitioner at XX Hospital, so I also went to see him.

#### 3.2.2 Perceived benefits from TCM

##### Improving sleep and fatigue

Most interviewees experienced improvements in sleep and fatigue after using TCM. They reported improved sleep quality, reduced sleep latency, and feeling more energetic and alert during everyday activities. TCM enabled patients to better cope with their condition, enhanced physical and mental well‐being, and an improved outlook on life.

“M” – The herbal medicine allowed me to sleep for 4‐5 h straight. Afterwards, I felt quite clear‐headed, and I didn't have to take a nap later in the day. Sleep quality was a lot better. I hadn't been able to sleep until 7 or 8 o'clock in years.

“A” – I used to take sleeping pills because I had trouble falling asleep. After taking TCM, my sleep returned to normal.

“I” – I would be extremely tired after chemotherapy. I would walk slower, and I sounded really tired. The herbal medicine raised my energy levels. My everyday life was a lot better. I could walk faster; I could do stuff again.

“P” – I definitely think herbal medicine helped. Even after chemotherapy, I still had the energy to play mahjong with my friends.

##### Relieving side effects of anticancer treatment

Relieving the side effects of anticancer treatment was a major benefit experienced by the interviewees. Some interviewees explicitly stated that using TCM was the only reason they could complete their chemotherapy regime. Some mentioned that TCM reduced the side effects of hormonal therapy.

“C” – I had some flu‐like symptoms after chemotherapy. I was given some drugs, which greatly helped, but they didn't get rid of them completely. However, I completely recovered in two days after using moxibustion and herbal medicine.

“I” – I really couldn't tolerate the doxorubicin side effects. I would tell all this to my TCM practitioner, and he would help me slowly relieve these symptoms. In fact, if it wasn't for his help, I don't think I would've been able to tolerate all 6 doses of doxorubicin.

“M” – My mouth was dry and bitter for a long time after chemotherapy; I couldn't even speak. TCM helped a lot, so I didn't have to always drink water.

“N” – I felt that my absorption worsened after chemotherapy, so I sought help from a TCM practitioner. I was quite thin, but I felt better after taking herbal medicine.

“E” – Acupuncture greatly helped with my swelling. After 10 acupuncture sessions, there was an obvious improvement in the oedema, and I had more muscle strength.

“O” – TCM reduced the hot flushes, sweating, and palpitations and helped with the skin pigmentation [toxicity of tamoxifen]. My stool was also back to normal.

##### Relieving stress and anxiety

TCM reduced stress and anxiety, leading to reduced emotional distress and improved mental health. “Feeling more stable” after taking herbal medicine was a common belief, although participants gave different reasons for how TCM lessened their emotional suffering.

“D” – In my head, I felt that herbal medicine could help prevent my cancer from relapsing, so I felt more peace of mind.

“M” – I was so stressed even after finishing all the treatments… My TCM practitioner gave me some medicine that suited me and helped ‘soothe my mood’.

“G” – My husband was very anxious for me. After I started taking TCM, he felt a lot more reassured, so I [also] felt less stressed.

#### 3.2.3 Barriers encountered when using TCM

##### Financial constraints

Although there were public and private TCM clinics, patients had to pay out‐of‐pocket for TCM advice, leading to a greater financial burden. Given the high costs associated, some interviewees were hesitant to continue TCM treatment.

“M” – The fee was such a burden… I initially went to the TCM practitioner my family members recommended, which was very expensive, and I couldn't afford it. The consultation fee was HKD500, with each dose costing HKD150‐170.

“C” – Seeing a private TCM clinic specialising in oncology was costly. It costs about HKD1000‐ HKD2000 per week.

“T” – I could buy TCM over the counter, like Ling Zhi supplements, but they were quite expensive, and I wasn't sure if they were useful.

##### Time constraints

The slow onset of TCM and the long boiling time of herbal medicine were cited as major limitations, with interviewees lamenting the time‐consuming nature of using herbal medicine. There was also the inconvenience of seeing a TCM practitioner multiple times per month on top of visits to the oncologist.

“O” – It was really difficult when I went back to work because you can't boil the herbs at work. I was also taking Western and herbal medicine simultaneously, and there needed to be a time gap between taking them, so scheduling my day was difficult with Chinese medicine and Western medicine.

“H” – I had to see the TCM practitioner at least twice a week. I needed to boil the herbs at home, once in the morning and evening, six times a week. Whereas for Western medicine, you only had to take a pill four times a day. It's time‐consuming having to boil the herbs.

“K” – It was quite slow, and you can't immediately see results…

“M” – My TCM practitioner is very popular. Every time, I need to wait for more than an hour.

##### Not recommended by oncologist

Some oncologists did not recommend TCM, though the degree of disapproval and reasoning for the objection varied. Concomitant use of TCM with standard therapy was not recommended.

“I” – My oncologist was quite against patients taking TCM while on Western medicine.

“S” – My doctor said TCM would raise the tumour markers when I check blood, so I stop TCM

#### 3.2.4 Recommendations on using TCM

All interviewees appraised the significant effectiveness of TCM for symptom relief and would recommend TCM as a complementary therapy to fellow survivors. Some also suggested integrating Western medicine and TCM in breast cancer treatment.

“N” – I recommend they seek TCM once they finish standard therapy. Those treatments are harmful to the body, and TCM can help regulate our health. Otherwise, it will be difficult to completely recover.

“H” – I would tell them to see a Western doctor first… After that, it's more appropriate to see a TCM practitioner because we're basically in a race against cancer. If we're slower than it, then it'll win.

“P” – I have recommended several friends with cancer to seek advice from TCM practitioners to improve their general health.

#### 3.2.5 Filling the care gap

The main drive for BCS to use TCM was to “fill the care gap” that Western medicine was unable to fulfil completely, which highlights the role TCM plays in a BCS's breast cancer journey. This “care gap” is difficult to quantify as it does not focus on medical data, survival or recurrence rates. Instead, the gap encapsulates the subjective and individual experiences and feelings of our BCS. They wished for an improvement in their persisting physical symptoms and emotional distress caused by conventional treatment but did not want to rely on Western medicine. They wanted to have a sense of control, better symptom relief and regain energy. This was facilitated by their trust that TCM was more natural and a milder form of treatment.

## 4 DISCUSSION

The theory of TCM is always a very individualised and personalised experience. It is not easy to quantify the experience of TCM usage. Our study used a qualitative approach focusing on BCS experiences, establishing a clearer understanding of the benefits of TCM. Our study found several important points: (i) most of the BCS decided to use TCM to “fill the care gap” which could not be fulfilled completely by conventional medicine; (ii) they wished to have a sense of control but not rely entirely on Western medicine; (iii) most felt an improvement in symptoms and regained energy after TCM usage; (iv) despite having barriers in using TCM, their positive experience still would encourage them to recommend TCM to other fellow survivors.

In our study, nearly all BCS who used TCM reported significant improvements in sleep quality, sleep latency, and cancer‐related fatigue. This finding was consistent with other studies where herbal medicine could effectively manage chronic fatigue and insomnia.^6^, ^30^, ^31^ Our participants also documented improved symptoms, such as constipation, hot flushes, oedema, palpitations, and dry mouth. With better symptom control, patients would have better psychological well‐being and higher confidence in self‐care. The optimisation of their overall well‐being would lead BCS to take a more active role in their cancer management.

Despite the benefits of TCM, financial and time constraints were highlighted as the main barriers to using TCM in our cohort. Our participants described a varying range of costs for a TCM consultation, depending on whether it is a public or private clinic, the quality and quantity of herbs used, and how long the TCM practitioner had been practising. There are some government subsidised TCM clinics, such as Kwong Wah Hospital and Tung Wah Hospital, but patients still need to pay for the herbs out‐of‐pocket. Therefore, this compounded the existing financial burden of BCS.

Participants often reported expressing disapproval or dismissal by their oncologists upon mentioning TCM. Most oncologists in China and Hong Kong do not encourage cancer patients to use TCM. Most treating oncologists state that the main reasons for not integrating TCM into routine clinical practice are: (i) TCM may interact with conventional treatment and (ii) a lack of adequate professional knowledge of the underpinning the biochemical profile of TCM.^32^ This level of caution is understandable as the TCM, especially Chinese herbal medicine, is inadequately studied, and there is insufficient data regarding herb‐drug interactions. In addition, the practice of polypharmacy in Chinese herbal medicine with individualised herbal formulations tailored to a patient's condition hinders the integration of TCM with oncology. Some herbal medicine may interact with anticancer therapies, affect organ function and even interfere with the pharmacokinetics (PK) and pharmacodynamics (PD) of cancer treatment.^33^, ^34^ Some commonly used herbs may affect the liver or renal function, while some may lower platelet count, prompting a temporary suspension of chemotherapy.^35^, ^36^, ^37^ More quantitative studies on the PK and PD of TCM would lessen oncologists' distrust and promote doctor‐patient discussion for the safe use of TCM.

Patient experience and subjective feelings are extremely difficult to quantify. The strength of the study was its qualitative design. Semi‐structured interviews enabled us to deeply explore the lived experiences of BCS on TCM usage, especially its interplay with their broader sociocultural context. Inductive analysis with grounded theory principles enabled us to derive a general theory for TCM use based on the views and experiences of our participants. However, it had several limitations. Firstly, participants of this study were recruited from a single institution and limited cancer survivor groups. Survivors are also more likely to remember and share positive, successful experiences with TCM than negative experiences. Secondly, criterion validity and reliability of the questionnaire in phase I were not assessed. However, its purpose was just to identify those who had experience with TCM usage and content validity was established to ensure this. Little to no questionnaire data was used in the qualitative analysis. Thirdly, interviews were conducted via video call instead of in‐person due to the COVID‐19 pandemic. Face‐to‐face interviews may be more meaningful.

## 5 CONCLUSIONS

This article presented an overview of the utilisation pattern, motivations, perception and benefits of TCM use among Chinese BCS in Hong Kong. TCM plays a supportive role by filling their unmet physical and emotional needs during and post‐treatment. Reduced symptom burden and the active pursuit of TCM led to improved well‐being and better coping and resilience.

This study contributed to an area of unmet clinical need and identified important areas for further research expanding on the understanding of TCM use in cancer survivors. Additional prospective and randomised controlled trials should focus on the efficacy and pharmacokinetic mechanism of TCM in combination with standard of care.

## AUTHORS' CONTRIBUTIONS

Conception and design: Siu‐Sing Leung, Yik‐Laam Hung, Wing‐Lok Chan. Data collection: Yik‐Laam Hung, Siu‐Sing Leung, Siu‐Ting Pamela Chiu, Pik‐Yi Li, Anson Chi‐On Kan, Chi‐Chung Lo, Sze‐Yuen Zeta Wong, Sze‐Lam Luk, Cheuk‐Lam Charlotte Lai. Analysis and interpretation of data: Yik‐Laam HUNG, Siu‐Sing Leung, Siu‐Ting Pamela Chiu, Pik‐Yi Li, Anson Chi‐On Kan, Chi‐Chung Lo, Sze‐Yuen Zeta Wong, Sze‐Lam Luk, Cheuk‐Lam Charlotte Lai. Manuscript writing and editing: Yik‐Laam Hung, Siu‐Sing Leung, Aya El Helali, Wing‐Lok Chan. Approval of final manuscript: Yik‐Laam Hung, Siu‐Sing Leung, Aya El Helali, Wing‐Lok Chan. All authors read and approved the final manuscript.

## FUNDING INFORMATION

This research received no external funding.

## CONFLICT OF INTEREST

The authors declare no conflicts of interest.

## ETHICS APPROVAL STATEMENT

This study was conducted in accordance with the Declaration of Helsinki and approved prior to study commencement by the Institutional Review Board of the University of Hong Kong/Hospital Authority Hong Kong West Cluster (HKU/HA HKW IRB), protocol code: UW 20–852.

## PATIENT CONSENT STATEMENT

Informed consent was obtained from all subjects involved in the study.

## Supporting information


Data S1

Data S2

## Data Availability

The de‐identified datasets generated that support the findings of this study are available upon reasonable request from the corresponding author Dr Wing‐lok Chan. The data are not publicly available due to privacy or ethical restrictions. Reuse if only permitted upon reasonable request.
