# Supplementary material for: Perceptions about traditional Chinese medicine use among Chinese breast cancer survivors: A qualitative study
Source: Cancer Med. 2022 Sep 8;12(2):1997–2007. doi: 10.1002/cam4.5046 (PMC9883569; doi:10.1002/cam4.5046)
Supplement: Supplementary file 1 — Data S1 [file CAM4-12-1997-s002.pdf]

Supplementary Material 1. Questionnaire

Please select the most appropriate option.

S1: Consent

Please select as appropriate.

i. I confirm that I have read and understood the information sheet for the above study and have had the opportunity to ask questions and consent to take part in this questionnaire.

- ❑ Yes

S2: Patient Background

1. **Age**
   - ❑ 18-29
   - ❑ 30-39
   - ❑ 40-49
   - ❑ 50-59
   - ❑ 60-69
   - ❑ 70 or above

2. **Highest Education Level Attained**
   - ❑ Primary school graduate
   - ❑ Junior secondary school graduate
   - ❑ Senior secondary school graduate
   - ❑ Bachelor's degree or higher

3. **Marital status**
   - ❑ Single
   - ❑ Married
   - ❑ Divorced
   - ❑ Widow
   - ❑ Others

4. **Occupation**
   - ❑ Professional/Manager/Administration
   - ❑ Businessman/Proprietor
   - ❑ White-collar worker
   - ❑ Blue-collar worker
   - ❑ Housewife
   - ❑ Full-time student
   - ❑ Unemployed
   - ❑ Retired
   - ❑ Other: Please specify

5. **Other malignancy unrelated to breast cancer:**
   - ❑ Yes – **Thank you for your participation!**
   - ❑ No

6. **Besides breast cancer, do you have any other chronic illnesses?**
   - ❑ No
   - ❑ Diabetes
   - ❑ Coronary Heart Disease
   - ❑ Hyperlipidaemia
   - ❑ Hypertension
   - ❑ Gout
   - ❑ Other: Please specify

<u>S3: Medical History</u>

Please select the most appropriate option.

7. **Age when you were diagnosed with breast cancer.**
   - ❑ 18-29
   - ❑ 30-39
   - ❑ 40-49
   - ❑ 50-59
   - ❑ 60-69
   - ❑ 70 or above

8. **What stage was your tumour when you were diagnosed?**
   - ❑ Stage 1
   - ❑ Stage 2
   - ❑ Stage 3
   - ❑ Stage 4
   - ❑ Unsure

9. **Which of the following treatments have you received? (Select all that apply)**
   - ❑ Surgical resection (e.g. Total mastectomy)
   - ❑ Chemotherapy
   - ❑ Radiotherapy
   - ❑ Targeted therapy
   - ❑ Hormonal therapy
   - ❑ Other: Please specify

10. **If you have undergone surgical resection, which of the following surgeries have you received?** (Select all that apply)
    - ❑ Total mastectomy
    - ❑ Breast-conservative surgery
    - ❑ Unsure
    - ❑ N/A

11. **How was your daily life being impacted by <u>surgical resection</u>? (If you have not undergone this treatment, please select N/A)**
    - ❑ Pain
    - ❑ Fatigue
    - ❑ Dizziness
    - ❑ Nausea/Vomiting
    - ❑ Sleeping problems
    - ❑ Difficulty remembering
    - ❑ No appetite
    - ❑ Dry mouth
    - ❑ Constantly stressed
    - ❑ Numbness/Sadness/Anxiety
    - ❑ N/A

12. **How was your daily life being impacted by <u>chemotherapy</u>? (If you have not undergone this treatment, please select N/A)**
    - ❑ Pain
    - ❑ Fatigue
    - ❑ Dizziness
    - ❑ Nausea/Vomiting
    - ❑ Sleeping problems
    - ❑ Difficulty remembering
    - ❑ No appetite
    - ❑ Dry mouth
    - ❑ Constantly stressed
    - ❑ Numbness/Sadness/Anxiety
    - ❑ N/A

13. **How was your daily life being impacted by <u>radiotherapy</u>? (If you have not undergone this treatment, please select N/A)**
    - ❑ Pain
    - ❑ Fatigue
    - ❑ Dizziness
    - ❑ Nausea/Vomiting
    - ❑ Sleeping problems
    - ❑ Difficulty remembering
    - ❑ No appetite
    - ❑ Dry mouth
    - ❑ Constantly stressed
    - ❑ Numbness/Sadness/Anxiety
    - ❑ N/A

14. **How was your daily life being impacted by <u>targeted therapy</u>? (If you have not undergone this treatment, please select N/A)**
    - ❑ Pain
    - ❑ Fatigue
    - ❑ Dizziness
    - ❑ Nausea/Vomiting
    - ❑ Sleeping problems
    - ❑ Difficulty remembering
    - ❑ No appetite
    - ❑ Dry mouth
    - ❑ Constantly stressed
    - ❑ Numbness/Sadness/Anxiety
    - ❑ N/A

15. **How was your daily life being impacted by <u>hormonal therapy</u>? (If you have not undergone this treatment, please select N/A)**
    - ❑ Pain
    - ❑ Fatigue
    - ❑ Dizziness
    - ❑ Nausea/Vomiting
    - ❑ Sleeping problems
    - ❑ Difficulty remembering
    - ❑ No appetite
    - ❑ Dry mouth
    - ❑ Constantly stressed
    - ❑ Numbness/Sadness/Anxiety
    - ❑ N/A

16. **How long have you recovered from breast cancer?**
    - ❑ 1-5 years
    - ❑ 6-10 years
    - ❑ 11-20 years
    - ❑ >20 years

17. **Year when you were diagnosed with breast cancer? (e.g. 2000) ________**

18. **Have you had any cancer recurrences?**
    - ❑ Yes
    - ❑ No

<u>S4: Use of Alternative Medicine (e.g. Orally-ingested treatments, Externally-applied treatments, Acupuncture, Cupping, Tui Na, Qi Gong, Tai Chi , Diet therapy, Music therapy, Aromatherapy, Hypnosis, Yoga)</u>

19. **Have you used alternative medicine in conjunction with your standard cancer treatment before?**
    - ❑ Yes **– Proceed to S5**
    - ❑ No **– Thank you very much for your participation**

S5: Use of Alternative Medicine (1)

20. **Was your use of alternative medicine related to breast cancer management?**
    - ❑ Related **– Proceed to S6**
    - ❑ Unrelated **– Proceed to S7**

S6: Use of Alternative Medicine (2)

21. **Which types of alternative medicine have you used? (Select all that apply)**
    - ❑ Aromatherapy
    - ❑ Traditional Chinese Medicine (TCM)
    - ❑ Naturopathy
    - ❑ Hypnosis
    - ❑ Yoga
    - ❑ Other: Please specify

22. **When did you start using alternative medicine therapies?**
    - ❑ I've always been using alternative medicine therapies
    - ❑ In conjunction with breast cancer treatment
    - ❑ After completion of breast cancer treatment
    - ❑ Other: Please specify

23. **How did you come to know about using alternative medicine?**
    - ❑ Always been using it
    - ❑ Recommended by friends/family
    - ❑ Recommended by doctor
    - ❑ From online sources
    - ❑ Other: Please specify

24. **In a week, how many times did you use alternative medicine therapies?**
    - ❑ <1
    - ❑ 1-3
    - ❑ 4-6
    - ❑ >7

S7: Use of Alternative Medicine (3)

25. **How effective do you think alternative medicine is to relieving the following side effects? (1- Very ineffective, 5- Very effective)**

	1	2	3	4	5
Pain	❑	❑	❑	❑	❑
Fatigue	❑	❑	❑	❑	❑
Dizziness	❑	❑	❑	❑	❑
Nausea/Vomiting	❑	❑	❑	❑	❑
Sleeping problems	❑	❑	❑	❑	❑
Difficulty remembering	❑	❑	❑	❑	❑
No appetite	❑	❑	❑	❑	❑
Dry mouth	❑	❑	❑	❑	❑
Constantly stressed	❑	❑	❑	❑	❑
Numbness/Sadness/Anxiety	❑	❑	❑	❑	❑

S8: Use of Traditional Chinese Medicine (TCM) (e.g. Orally-ingested treatments, Externally-applied treatments, Acupuncture, Cupping, Tui Na, Qi Gong, Tai Chi , Diet therapy)

26. **Have you used TCM?**
    - ❑ Yes – **Proceed to S9**
    - ❑ No – **Proceed to S11**

S9: Use of Traditional Chinese Medicine (1)

27. **Was your use of TCM related to breast cancer management?**
    - ❑ Related – **Proceed to S10**
    - ❑ Unrelated – **Proceed to S11**

S10: Use of Traditional Chinese Medicine (2)

28. **How did you come to know about using TCM?**
    - ❑ I've always been using TCM
    - ❑ Recommended by friends or family
    - ❑ Recommended by doctors
    - ❑ From online sources
    - ❑ Other: Please specify

29. **Why did you use TCM?**
    - ❑ To take care of the body
    - ❑ Treating symptoms
    - ❑ Prevent recurrence
    - ❑ Relatively cheap
    - ❑ Other: Please specify

30. **When did you start using TCM?**
    - ❑ I've always been using TCM
    - ❑ During standard treatment
    - ❑ After standard treatment
    - ❑ Other: Please specify

31. **At which stage of your therapy did you use TCM?**
    - ❑ Surgery
    - ❑ Chemotherapy
    - ❑ Radiotherapy
    - ❑ Target therapy
    - ❑ Hormonal therapy
    - ❑ N/A
    - ❑ Other: Please specify

32. **Which TCM practice have you used during standard treatment?** (**Select all that apply**)
    - ❑ Orally-ingested treatments (e.g. Herbal teas)
    - ❑ Externally-applied treatments (e.g. Herbal pastes)
    - ❑ Acupuncture
    - ❑ Cupping
    - ❑ Tui Na
    - ❑ Qi Gong
    - ❑ Tai Chi
    - ❑ Diet therapy
    - ❑ N/A
    - ❑ Other: Please specify

33. **Which TCM practice have you used after recovery? (select all that applies)**
    - ❑ Orally-ingested treatments (e.g. Herbal teas)
    - ❑ Externally-applied treatments (e.g. Herbal pastes)
    - ❑ Acupuncture
    - ❑ Cupping
    - ❑ Tui Na
    - ❑ Qi Gong
    - ❑ Tai Chi
    - ❑ Diet therapy
    - ❑ N/A
    - ❑ Other: Fill in

34. **How long did you use TCM?**

- ❑ <1 month
- ❑ 1-6 months
- ❑ 7-12 months
- ❑ >1 year
- ❑ N/A

35. **In a week, how many times did you use TCM?**

- ❑ <1
- ❑ 1-3
- ❑ 4-6
- ❑ >7
- ❑ N/A

S11: Use of Traditional Chinese Medicine (3)

36. **How effective do you think the following TCM are in relieving symptoms? (1- Very ineffective, 5- Very effective)**

	1	2	3	4	5
Orally-ingested treatments (e.g. Herbal teas)	❑	❑	❑	❑	❑
Externally-applied treatments (e.g. Herbal pastes)	❑	❑	❑	❑	❑
Acupuncture	❑	❑	❑	❑	❑
Cupping	❑	❑	❑	❑	❑
Tui Na	❑	❑	❑	❑	❑
Qi Gong	❑	❑	❑	❑	❑
Tai Chi	❑	❑	❑	❑	❑
Diet therapy	❑	❑	❑	❑	❑

37. **How effective do you think TCM is in relieving the following symptoms? (1 - Very ineffective, 5- Very effective)**

	1	2	3	4	5
Pain	❑	❑	❑	❑	❑
Fatigue	❑	❑	❑	❑	❑
Dizziness	❑	❑	❑	❑	❑
Nausea/Vomiting	❑	❑	❑	❑	❑
Sleeping problems	❑	❑	❑	❑	❑
Difficulty remembering	❑	❑	❑	❑	❑
No appetite	❑	❑	❑	❑	❑
Dry mouth	❑	❑	❑	❑	❑
Constantly stressed	❑	❑	❑	❑	❑
Numbness/Sadness/Anxiety	❑	❑	❑	❑	❑

38. **From a scale of 1-5, would you encourage other breast cancer patients to use TCM? (1 - Very discouraged, 5 - Very encouraged)**

**1**<br>Very discouraged	**2**	**3**	**4**	**5**<br>Very encouraged
❑	❑	❑	❑	❑

39. **What do you believe are the limitations in using TCM?** (Select all that apply)
    - ❑ Too expensive
    - ❑ Discouraged by doctor
    - ❑ No apparent effect
    - ❑ Requires a long time to be effective
    - ❑ No scientific proof on its efficacy
    - ❑ Lack strict regulation on its prescription
    - ❑ Do not believe there are any limitations
    - ❑ Other: Please specify

<u>S12: Further Interviews</u>

Thank you for completing the questionnaire. We would like to conduct an interview to further understand your experience in using TCM in conjunction with standard breast cancer treatment.

40. **Would you like to participate in the interview?**
    - ❑ Yes – **Proceed to S13**
    - ❑ No – **Thank you for participating in the questionnaire**
