# Supplementary material for: Perceptions about traditional Chinese medicine use among Chinese breast cancer survivors: A qualitative study
Source: Cancer Med. 2022 Sep 8;12(2):1997–2007. doi: 10.1002/cam4.5046 (PMC9883569; doi:10.1002/cam4.5046)
Supplement: Supplementary file 2 — Data S2 [file CAM4-12-1997-s001.pdf]

Supplementary Material 2. Interview Guide

Interview Guide

1. What impact did your diagnosis have on your Life?
   - *Probe*—Physical, spiritual/emotional, financial, work/home life

2. Had you used TCM before you were diagnosed?

3. If you hadn't used it before, how did you find out about TCM?

4. Did you use TCM during anti-cancer treatment? Will you consider doing TCM during the anti-cancer treatment period?

5. What was the reason for you to start TCM?

6. At what stage did you use TCM?

7. *What type of TCM therapies did you seek/receive?

8. At what aspect do you think TCM can help you?
   - *Probe*—Physical(Symptom relieving, side effect relieving), spiritual/emotional

9. What do you experience as the benefits of TCM therapies?
   - *Probe*—Tell me a little more about it?

10. What do you experience as the limitations of TCM therapies?
    - *Probe*—Tell me a little more about it?

11. Did you experience any barriers in using TCM?
    - *Probe*—Time, distance, financial?

12. Did your perception about TCM change after you used TCM? What changed?

13. What advice would you give someone, newly diagnosed with breast cancer, based on your experience?

14. Will you recommend TCM to our breast cancer survivors? Why?
    - *Probe*—Tell me a little more about it?

15. Do you have any other thoughts about breast cancer you would like to share?
